# Permafrost Degradation and Subsidence Observations during a Controlled Warming Experiment

**DOI:** 10.1038/s41598-018-29292-y

**Published:** 2018-07-19

**Authors:** Anna M. Wagner, Nathaniel J. Lindsey, Shan Dou, Arthur Gelvin, Stephanie Saari, Christopher Williams, Ian Ekblaw, Craig Ulrich, Sharon Borglin, Alejandro Morales, Jonathan Ajo-Franklin

**Affiliations:** 1 U.S. Army Cold Regions Research & Engineering Laboratory (CRREL), Fairbanks, AK USA; 2 Earth and Planetary Sciences Department, University of California, Berkeley, CA USA; 3 Lawrence Berkeley National Laboratory, Berkeley, CA USA; 4 U.S. Army Cold Regions Research & Engineering Laboratory (CRREL), Hanover, NH USA

## Abstract

Global climate change has resulted in a warmer Arctic, with projections indicating accelerated modifications to permafrost in the near future. The thermal, hydrological, and mechanical physics of permafrost thaw have been hypothesized to couple in a complex fashion but data collection efforts to study these feedbacks in the field have been limited. As a result, laboratory and numerical models have largely outpaced field calibration datasets. We present the design, execution, and initial results from the first decameter-scale controlled thawing experiment, targeting coupled thermal/mechanical response, particularly the temporal sequence of surface subsidence relative to permafrost degradation at depth. The warming test was conducted in Fairbanks, AK, and utilized an array of in-ground heaters to induce thaw of a ~11 × 13 × 1.5 m soil volume over 63 days. The 4-D temperature evolution demonstrated that the depth to permafrost lowered 1 m during the experiment. The resulting thaw-induced surface deformation was ~10 cm as observed using a combination of measurement techniques. Surface deformation occurred over a smaller spatial domain than the full thawed volume, suggesting that gradients in cryotexture and ice content were significant. Our experiment provides the first large field calibration dataset for multiphysics thaw models.

## Introduction

Recent and forecasted global climate change trends have led to observations and predictions of increased thermal degradation of frozen ground^1,2^. At Earth’s high latitudes, this transition threatens one of the largest carbon sinks on the planet, an estimated 2.7 B metric tons of frozen organic soil stored in permafrost^3^. Additionally, permafrost thaw modifies landscapes, affects ecological and biological systems, and destabilizes arctic infrastructure, which has led the United Nations Environmental Program to identify thawing of permafrost as one of the most significant yet least studied environmental hazards^4–8^. More than 40% of Earth’s high latitude permafrost will be lost by 2100^9^ where damage to future arctic infrastructure is of great concern. Permafrost environments are a unique setting for built infrastructure where minor surface or subsurface thermal perturbations can lead to significant substrate consolidation. Permafrost warming and thaw subsidence can occur slowly as prograde thaw fronts deepen the seasonally-unfrozen “active” layer year-upon-year, or rapidly as thermokarst (surface subsidence) features that degrade deep zones of previously ice-rich soil over weeks to months^5,10^. An individual thaw zone may only span ten meters, but tessellate over multiple watersheds^11^. Structural foundation damage and even catastrophic failure of roads, bridges, and runways due to permafrost degradation have been documented across Canada, Alaska, and northern portions of Europe and Asia^12–15^. One cumulative estimate of the expense from climate-related damage to Alaskan public infrastructure for the period 2015 – 2099 is $5.5B^16^. Infrastructure failure often occurs without precursory observations of the escalating risk, because the pan-arctic extent of the hazard and the resolution required to “catch” any single nascent thaw zone is ill-posed for conventional monitoring strategies. Ideally, infrastructure would involve sensing network elements to provide “awareness” of environmental changes that could provide warnings to infrastructure managers before failure.

In order to understand the role of permafrost thaw in future trends of global climate change and the related arctic infrastructure hazard, it is necessary to develop numerical, laboratory, and field-based approaches that are capable of capturing the relevant physical processes, including important spatial and temporal dynamics^17–19^. For example, understanding how soil bulk and shear modulus evolve as permafrost thaws could facilitate development of a permafrost thaw early warning system using dense seismic sensors along critical infrastructure like railways and pipelines. Predicting how effective stress and heat transport will evolve at an interface in low permeability silt and clay given a possible range of environmental conditions, and specifically how these processes upscale to a useful global climate model input is another important topic of research^3,20^. Complicated thermal, hydrological, and mechanical feedbacks with multiphase freeze-thaw dynamics have been hypothesized using realistic parameters and timescales^21–24^, yet field-scale calibration is difficult resulting in that laboratory and numerical studies presently outpace instrumented observational datasets.

The majority of permafrost thaw observations are campaign-based or utilize historical remote sensing datasets. This approach to field calibration has two challenges. First, although remote sensing and ergodic field campaigns have generated a set of geophysical observations that can be used to test hypotheses^25–30^, the massive scale demanded by atmospheric models and available parameter space involved in coupled thermo-hydraulic-mechanical (THM) feedbacks requires development of innovative ways to effectively capture sub-grid cell processes and calibrate all of the possible models. A second problem with the present limited inventory is that the rate and impacts of anthropogenically-driven warming still require decades of observation to conclusively characterize a process; surface subsidence is a difficult target due to superimposed seasonal cycles. Examination of the impact of warming magnitude and timing on processes is also complicated by the coupling of temperature gradients with secondary variables (e.g. soil moisture) across time-for-space comparison transects.

These limitations have motivated development of a variety of artificial warming approaches to allow exploration of the impact of temperature on relevant biological, biogeochemical, and physical properties at a range of length and time scales. A range of studies examining surficial alteration, particularly those involving small (meter to sub-meter) plots have utilized infrared lamps to induce controlled heating on the order of 1–10 °C^31^. Other warming experiments have explored the use of snow fences to provide semi-controlled warming through use of winter snow accumulation as an insulating driver. Starting with the work of Hinkel and Hurd Jr^32^, a sequence of studies have used snow fences to increase permafrost temperature by as much as 14 °C at seasonal peaks, in some cases generating subsidence on the order of 10–20 cm over 8 years of installation. Recently, the CiPHER project^33–35^ combined snow fence treatments, spring snow removal, and surface warming to provide year-round modifications to soil temperature. Despite their success, several limitations of snow fence alterations exist, including the substantial duration of the resulting experiments (multi-seasonal, often spanning 3–5 years), complicated seasonality of the driving force (winter, relative to snow depth), and coupled impacts on hydrology. For this study we instead adopted a strategy that was originally proposed by Hanson *et al*.^36,37^, Krassovski *et al*.^38^ and Wagner *et al*.^39^ but had not been fully implemented. Our approach utilized an array of subsurface electric heaters installed in a tight grid to manipulate subsurface temperature over a decameter scale during a single field season. The advantage of this approach is the ability to precisely probe the coupling between energy flux, thermal alteration, and resulting mechanical alteration or permafrost degradation over short durations. Although natural permafrost degradation usually occurs more slowly, a controlled-warming experiment allows one to accelerate the near-surface environment and thus quantify the important relationship between subsurface thaw and surface topography, capture the geophysical signatures of the advanced thaw process, and test novel sensing modalities such as low-cost instruments, distributed fiber-optics, and remote imaging methods which might someday be combined in a permafrost early warning system.

In 2016, we performed a permafrost controlled-warming experiment in Fairbanks, Alaska and monitored changes using variety of traditional and novel remote sensing, geophysical, and hydrological techniques to capture the coupling between deep soil thermal perturbations and surface subsidence. In this paper we present results from borehole thermistor and thermocouple monitoring, surface Electronic Distance Measurement (EDM) measurements, differential GPS (DGPS), and campaign LiDAR. Using this dataset, we show that this class of artificial warming array can be utilized for (a) extensive soil column heating over short durations, (b) permafrost table ablation representative of decades of warming, and (c) surface subsidence over the decameter range of lateral extent. This capability has immediate utility for validating coupled thermal-mechanical models of permafrost, testing geotechnical monitoring systems, and exploring the biogeochemical impacts of warming scenarios.

## Methods

### Site History and Background Data

**Figure 1:**
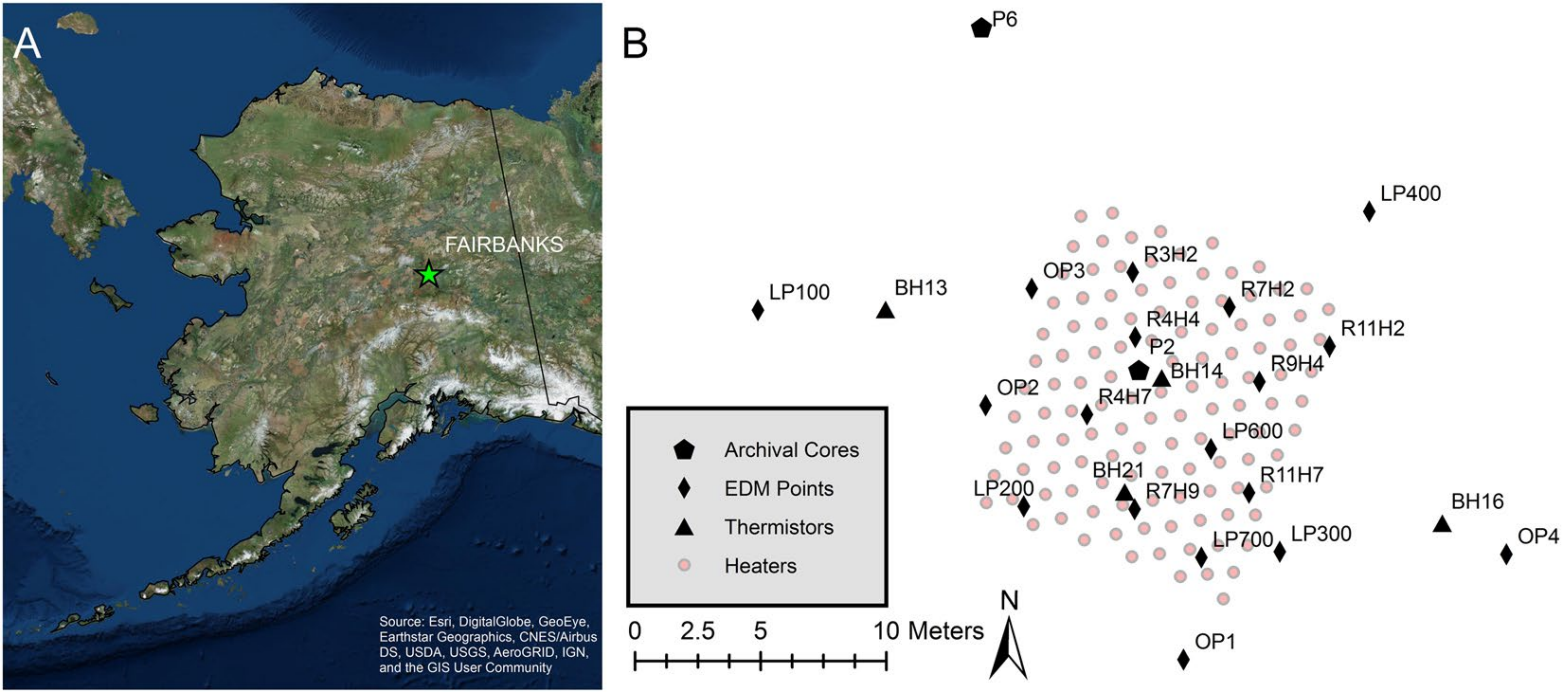
Site Overview. Panel A shows site location in Alaska. Panel B depicts heater layout (red circles), archival borehole locations (black hexagons), borehole temperature monitoring locations (black triangles), and EDM monument locations (black diamonds). The figure was generated using ArcGIS (ArcMap 10.5) software http://desktop.arcgis.com/en/arcmap/.

Our site is located in Fairbanks, Alaska, in the interior region of Alaska at 64.80°N; Fairbanks has a continental sub-Arctic climate with a mean annual air temperature of −2.9 °C (1971–2000) with a range of −23.2 °C to 16.9 °C^40^ and is characterized by discontinuous permafrost on the cusp of stability. Our experiment was conducted at the Fairbanks Permafrost Experiment Station (Fig. 1), located about 4 km northeast of Fairbanks on Farmers Loop Road, which was established in 1945 by the Field Operations Branch of the Permafrost Division of the U.S. Corps of Engineers St. Paul District and currently operated by the Cold Regions Research Engineering Laboratory (CRREL). The site consists of both ice-rich and discontinuous warm permafrost soils. Tan silts are encountered at the surface and grey silt are located at depths exceeding 1.4 m^41^. Soil grain size analysis of cores collected at multiple locations revealed the site was dominated by relatively homogeneous silt loams with a small fraction of very fine sand (see Table S1, Fig. S1) at all sampled depths. X-ray computed tomography (CT) analysis of recent cores has shown laminar ice features and some disseminated ice in silt sections (see Fig. S2). Soil saturation is normally within 0.3 m or less of the surface and moisture contents range from 26% to 41% by mass for silts with peat down to a depth of 9.5 m at the Linell plots^41^. Modern permafrost table depths vary across the heating plot, ranging from 1.5 m to the east to ~6 m to the west; this variability is due to historical changes in vegetation coverage.

### Heater array design, installation, and heating protocol

**Figure 2:**
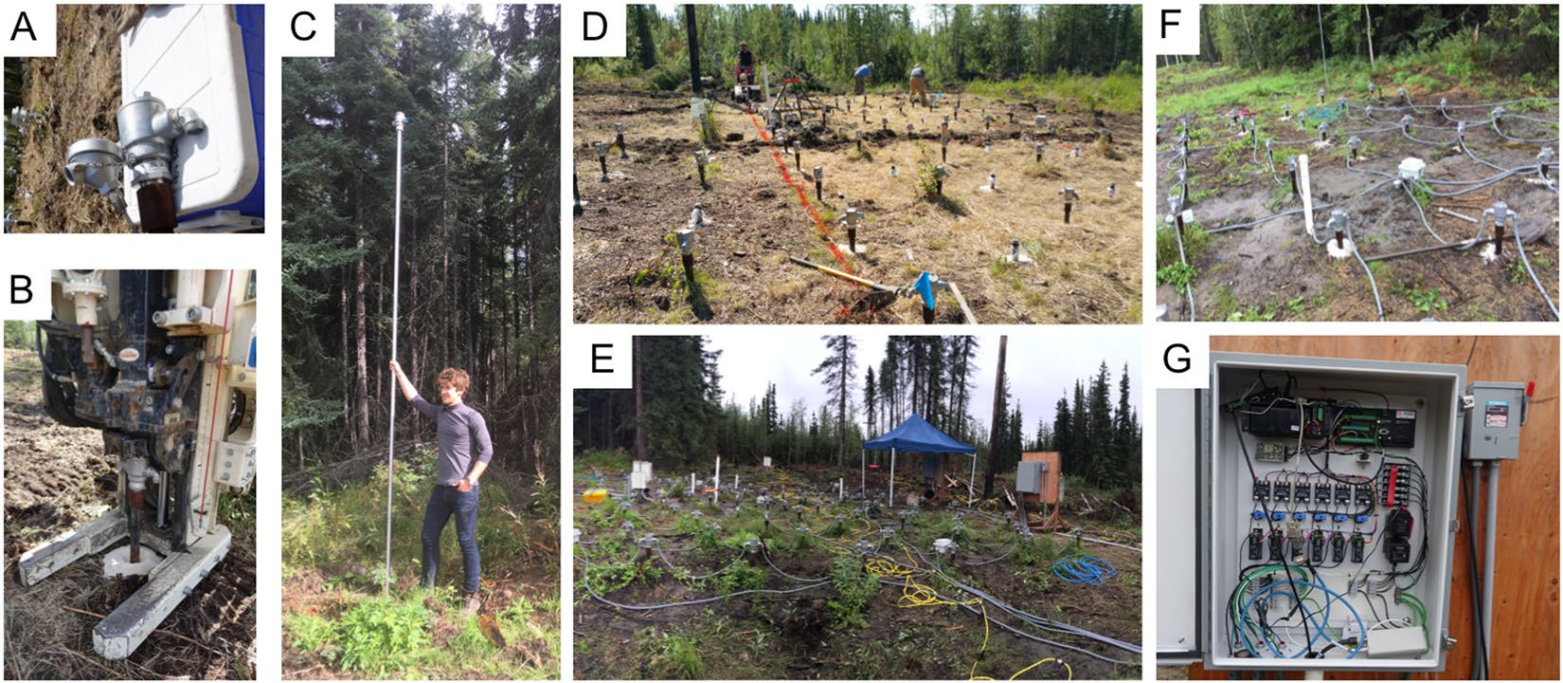
Photographs of the heating system installation and components: Panel A depicts heater head assembly. Panel B depicts push install of heater casing. Panel C shows full heater assembly. Panel D shows array after initial installation. Panel E shows site after completion. Panel F shows serial heater wiring configuration. Panel G shows heater control system.

The study site was previously disturbed and cleared for an ecosystem prototype warming installation system in 2010. The objective of that study was to develop an in-ground warming system that could artificially simulate soil-warming^39^. Components of the present heating system are shown in Fig. 2. The heating elements were re-purposed and reinstalled for this study during the summer of 2016. The vertical heaters (Indeeco Industries) had an outer diameter of 33.3 mm and measured 4 m in length. Each heater used 120 V, 50/60 Hz power and injected 60 W continuously into the ground. Only the bottom 1 m section of each element was actively heating, by design.

Figure 1, panel B and Fig. 2 panels D and F show the geometry and installation pattern of the heating array. A total of 121 heaters were emplaced in 11 rows over a 133 m^2^ area (10.5 m × 12.7 m). Each row was installed with an offset resulting in a distance between each heater of about 1.2 m. The heaters were installed using a track-mounted, direct-push drill rig (GeoProbe 7822) where a 5.7 cm solid-stem drill pipe was first pushed into the ground until the required depth was reached, and then the drill pipe was removed. The 42.2 mm outer diameter steel heater casings (1 1/4″ schedule 40) were then placed in the resulting holes and pushed with the drill rig to the required depth leaving about 0.15 m exposed above the ground surface. Figure 2 panel B shows the push process. Silica sand was used to backfill between the native soil and the heater casings. When all the heater casings had been installed the heater elements were placed in the heater casing. The heaters were wired in series using 12-gauge solid wire and the wire was protected using armored flexible conduit. Each row had a total of 11 heaters and they were wired into a single circuit that resulted in 11 circuits. All circuits were wired into two breaker boxes (Fig. 2 panel G) and the heaters were monitored using standard data loggers (Campbell Scientific CR1000). The heaters were turned on August 5, 2016 and subsurface heating continued through November 11, 2016.

### Temperature Monitoring

The heater array temperature and soil temperature at a few selected depths and locations were recorded using thermocouples (W.H. Cooke & Co., Inc.; see Fig. 1B for locations). Eight thermocouples were attached to heater casings throughout the plot during drilling operations. Soil temperatures were recorded at six locations throughout the heated area using multi-sensor T-type thermocouple strings (W.H. Cooke & Co., Inc). Near-surface soil temperatures were recorded at 0, 0.05 m, 0.20 m, 0.40 m, 0.80 m, 1.60 m, and 2.00 m. Schedule 80 PVC pipe with a nominal diameter of 19 mm, were installed to provide locations for more precise temperature measurement by way of thermistor strings. Two 6-m long PVC pipes (BH14, and BH21) were installed in the heated section of the plot and four 10-m PVCs were placed outside the heated area. One of the PVCs outside the plot was installed in the shallow permafrost to the east of the heated area (BH16) and three strings (BH13, BH19, and BH20) were installed to the west of the heated plot approximately 6 to 7 m from the heaters. The four PVCs outside the heated area were remaining infrastructure from the ecosystem heating prototype experiment. Soil temperatures outside the heated area were measured at 2, 4, 6, and 9 m depth and the soil temperatures inside the heated area was recorded every 0.5 m to a depth of 5.5 (BH14) and 6 m (BH21). Temperature probes (Campbell Scientific 107) and loggers (Campbell Scientific CR1000) were used to measure the soil temperatures in the PVC pipes at a temporal interval of 10 min throughout the experiment.

### Mechanical Deformation Monitoring

In order to examine the impact of permafrost thaw on surface deformation, the topography of the ground surface was measured throughout the heating experiment using a combination of techniques. Traditional two-point electronic distance measurements (EDM) were acquired using a manual total station (Leica Flexline TS06) and a survey-grade global positioning system (Trimble R8). Survey control was established with a static survey using the GPS, for an occupation time of a minimum of two hours, and receiving corrections using the Online Positioning User Service (OPUS) for stated accuracies of <2 cm. Benchmarks were established throughout the site (see Fig. 1) that were used both during the EDM and DGPS surveys. In addition to the benchmark surveys the heater elements and ground surface at the heater/ground surface boundaries were also surveyed throughout the heating experiment.

Prior to starting the heating experiment, a terrestrial based LiDAR scanner was used to capture the initial ground topography on August 5th, 2016. The LiDAR surveys were collected using a Leica ScanStation C10 and were performed approximately once a week (depending on weather), for a total of nine scans during the heating phase, with the final scan acquired on October 7th, 2016. A minimum of four scan positions were occupied to ensure complete coverage of the heater plot.

## Results

As mentioned previously, the heating experiment was conducted over three months from 05-Aug to 11-Nov-2016. The heating array functioned as expected over this time period with no major shutdown periods except for a brief power outage. Supplemental Fig. S3 shows total energy usage for the 11 heater sub-arrays which varies between 6.5 and 6.8 kW over the course of the experiment. Tight monitoring of current draw and heater surface temperature provided excellent energy flux constraints for coupled thermomechanical modeling; heater casing surface temperatures reached ~45 °C immediately before the end of the experiment (Fig. S3, lower panel).

**Figure 3:**
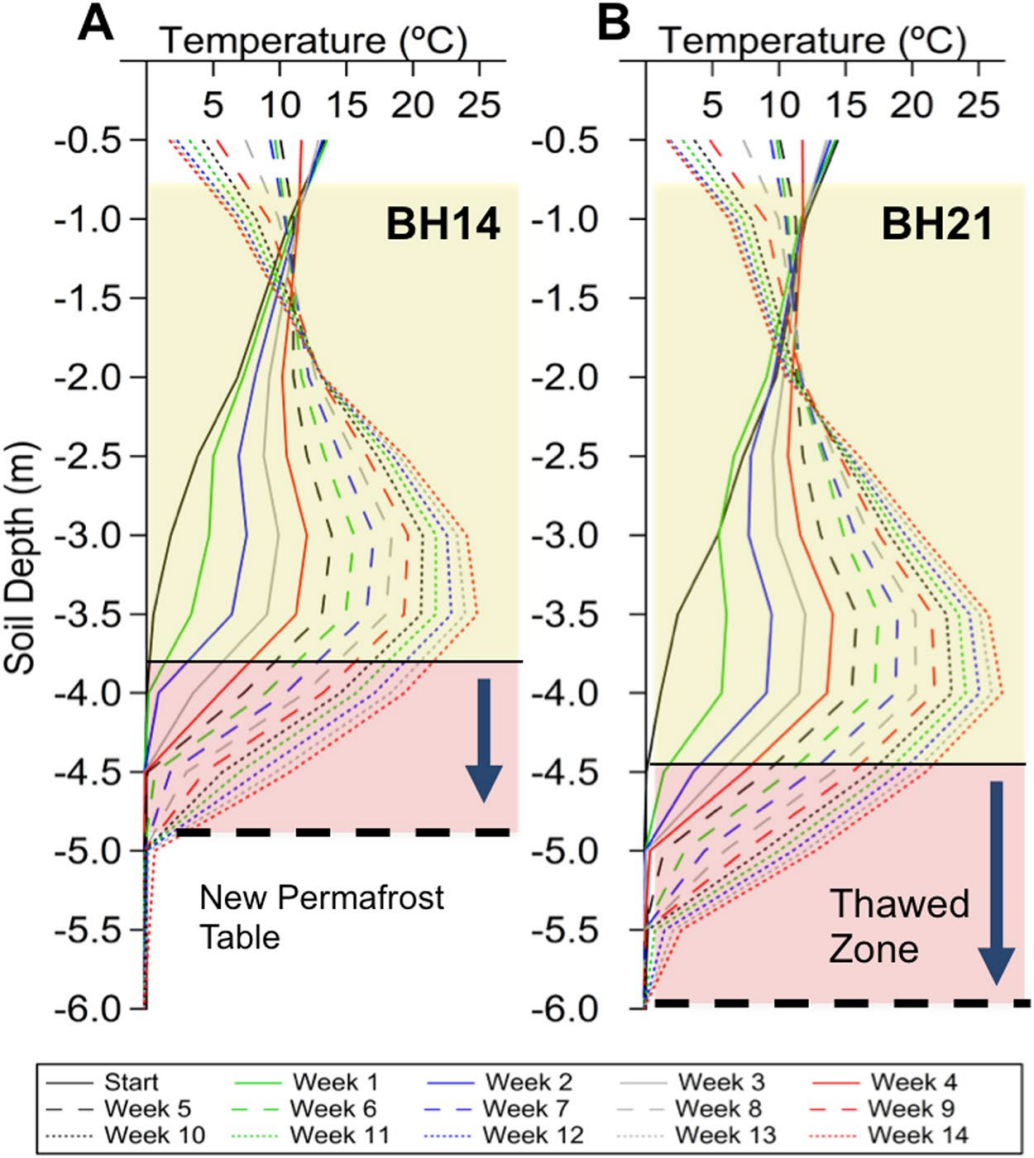
Thermal history during the heating experiment. Panel A shows the temperature history as measured using the borehole thermistor array at BH14. Depth-dependent temperature curves are shown for each week from baseline to the experiment conclusion. The descent of the permafrost table boundary (0 °C) is shown in pink highlight. Panel B depicts the same results for thermistor array BH21.

Figure 3 shows downhole temperature acquired during each week of the experiment in monitoring boreholes BH14 and BH21 in the center and southern side of the heating plot respectively. As expected, the heating protocol effectively increased temperature at monitoring locations between the heating elements; temperature values at the heating interval increased from slightly below 0 to 25 °C over the course of 14 weeks of heating (Fig. 3 and Fig. S3). The applied thermal load deepened the permafrost table between 1.25 and 1.5 m, depending on measurement location as can be seen by the highlighted regions in Fig. 3. Interestingly, the thermal load was not visible at the surface due to the concurrent seasonal cooling, visible at depths shallower than 2 m. Heating was not observed in zones of shallow permafrost off the plot. Monitoring borehole BH16, located ~7 m west of the heating plot, remained below 0 °C during the heating pilot at 2, 4, and 6 m depths as can be seen in Fig. S4. Advective heat transport was visible off the plot at unfrozen downslope monitoring locations (Fig. S4), likely driven by hydrologic coupling to thermal transport.

**Figure 4:**
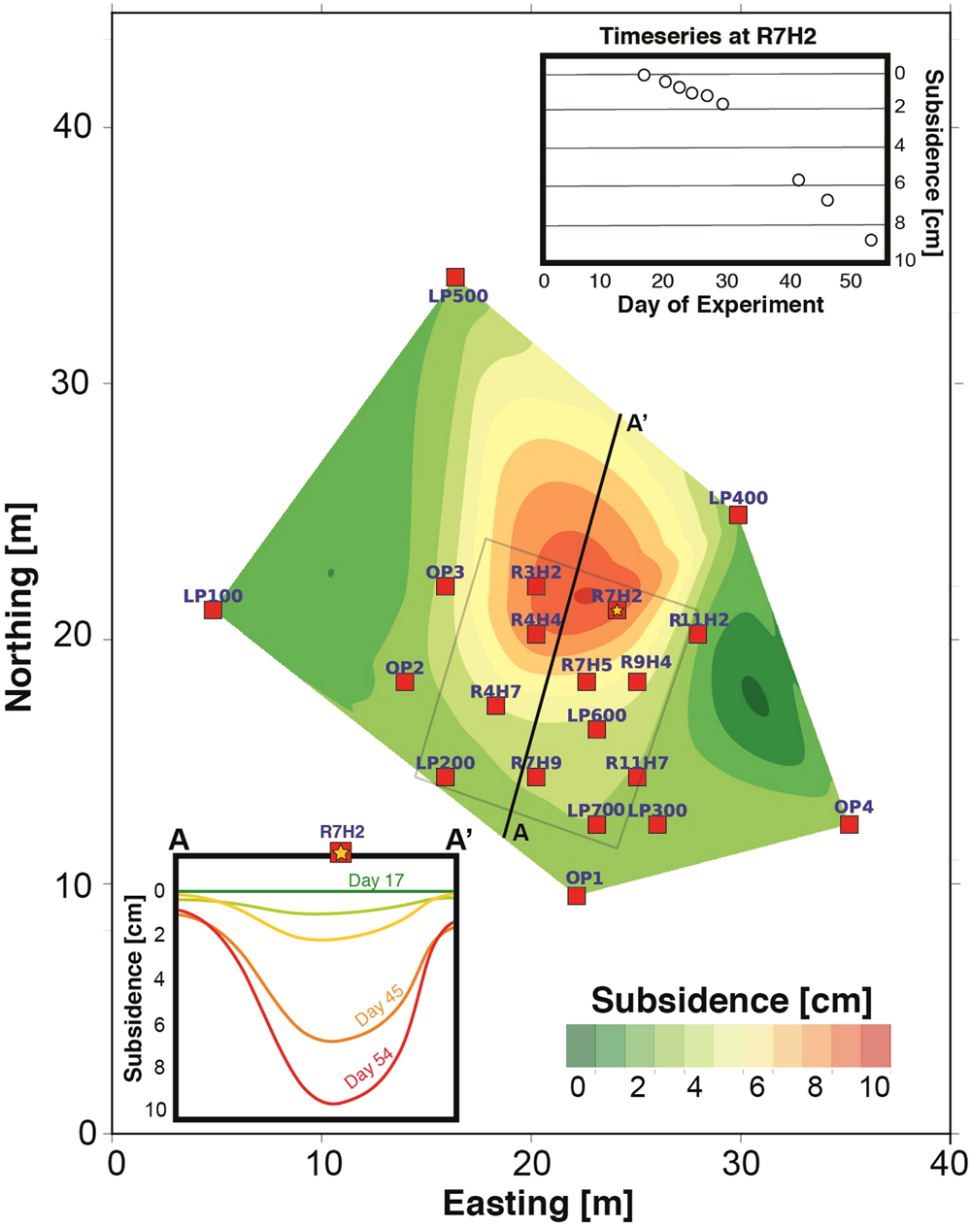
Electronic Distance Measuring (EDM) survey results for surface subsidence during the heating experiment. Central plot depicts difference in surface elevation between baseline and week 14. Colors are interpolated from measurements at fixed monuments (red squares). Top right panel shows time-history of subsidence measurements at monument R7H2. Bottom left panel shows a cross-section of elevation change from points A to A’.

Ablation of the permafrost table resulted in localized surface subsidence. Figure 4 shows the results of sequential EDM measurements quantifying the change in surface elevation over the course of the experiment. The central panel shows the difference between the baseline elevation and the elevation measurements at day 54 of the heating experiment. Measurements were conducted at surface monuments shown as red squares; the resulting differences were interpolated. As can be seen, subsidence with magnitudes between 4 and 10 cm were observed, with the largest deformation on the northern border of the heating plot. The measured onset of surface subsidence at station R7H2 was approximately 20 days after heating initiation (Fig. 4, panel A) and exhibited a nearly linear rate, potentially suggesting ablation of distributed rather than highly localized ice.

**Figure 5:**
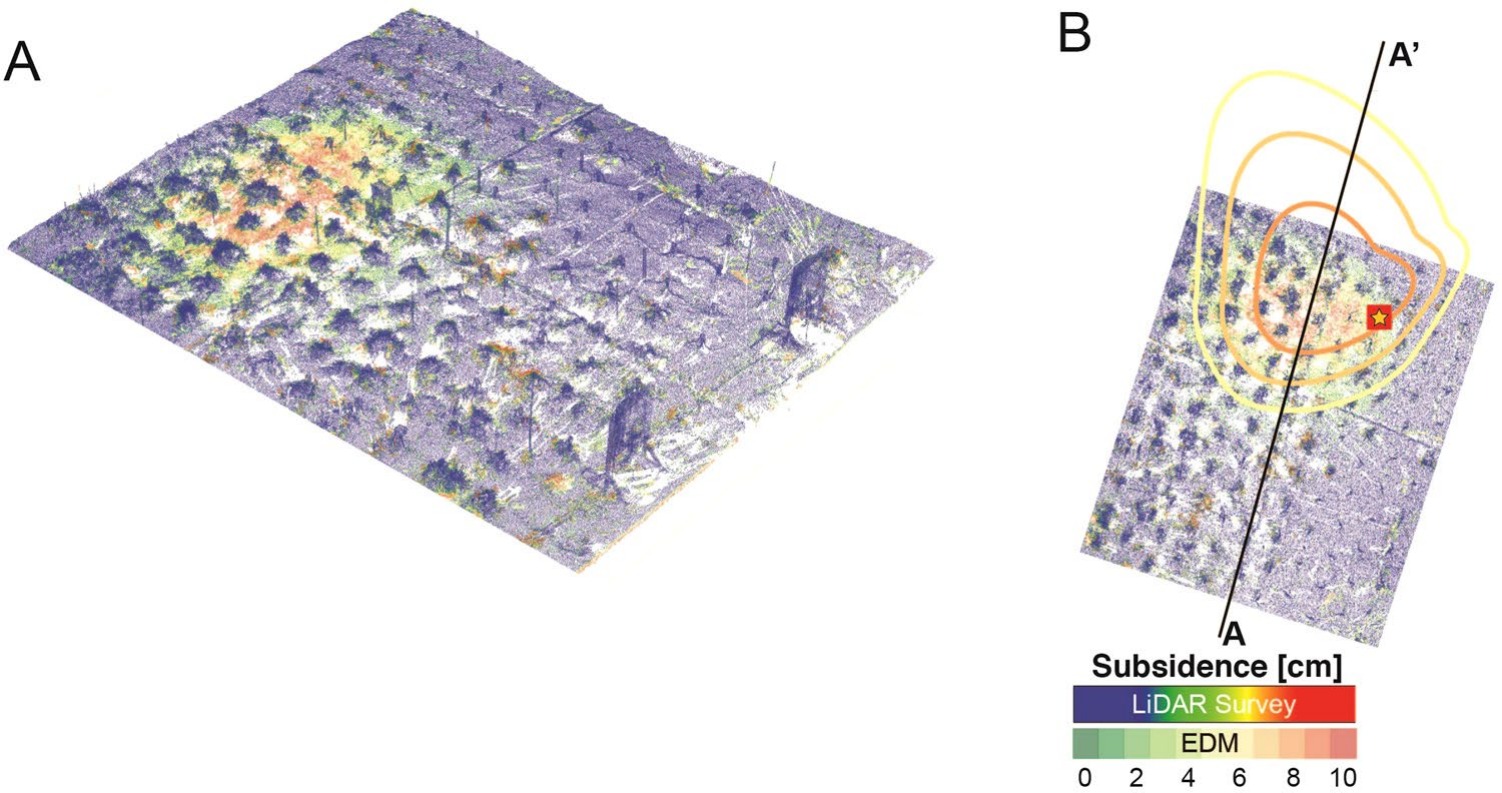
Differential LiDAR measurements spanning the heating experiment. Panel A shows a perspective view of the difference in surface elevation between baseline and week 14 as measured by LiDAR over the heater plot. Panel B shows a top view of the same dataset with superimposed contours showing co-located EDM subsidence measurements.

A higher resolution map of the same surface subsidence process was provided by repeat LiDAR surveys. The dense point clouds from the baseline survey on 05-Aug-2016 and the final survey on 07-Oct-2016 were differenced using an open-source software tool (CloudCompare) to yield the image shown in Fig. 5 (panel A). Isolated points with large positive differences, corresponding to growing vegetation, were removed before point cloud visualization. The zone of subsidence appears to be localized to the heating plot, but again on the northern section. The magnitude of the EDM and LiDAR-derived vertical deformations are quite similar, showing maximum values of approximately 10 cm. Figure 5 panel B shows the tight spatial correspondence of the LiDAR (colored) and EDM (contoured) subsidence measurements. Note that in Figs 4 and 5, the northward extent of the subsidence zone is slightly exaggerated to the north because of the larger spacing of EDM benchmark measurements between the heater plot and monuments LP400 and LP500, a linear interpolation artifact that is corrected in the dense LiDAR image.

## Discussion

We have demonstrated that dense borehole heater arrays can be utilized to effectively thaw large zones of permafrost and trigger surface subsidence at a scale relevant to understand civil infrastructure performance, evaluate thaw hazard monitoring approaches, and validate numerical models of coupled processes in permafrost environments. While prior studies have generated subsidence through snow fences over multiple seasons, we believe that this is the first study to provide a practical approach for generating decameter permafrost degradation features over a single field season, thus opening opportunities for rapid controlled evaluation of such processes. The heater array design, combining industrial resistive heaters housed in rugged well casings, was straightforward to install using a small direct-push rig and proved reliable over extended periods.

An outstanding question resulting from the early stage of this work is the concentration and asymmetry of the observed surface subsidence. The dominant known gradient at the site is the dip of the permafrost table, which deepens to the west and is close to the surface immediately to the east of the warming plot. We hypothesize that lateral variations in ice content, shown to be layered lenticular from nearby cores, generated this heterogeneity. Computed tomography (CT) scans of cores acquired at location OP4 (Fig. S2) reveal both laminar and more concentrated regions of ice-rich material; coring at alternate off-plot locations will be required to confirm the ice variability hypothesis.

**Figure 6:**
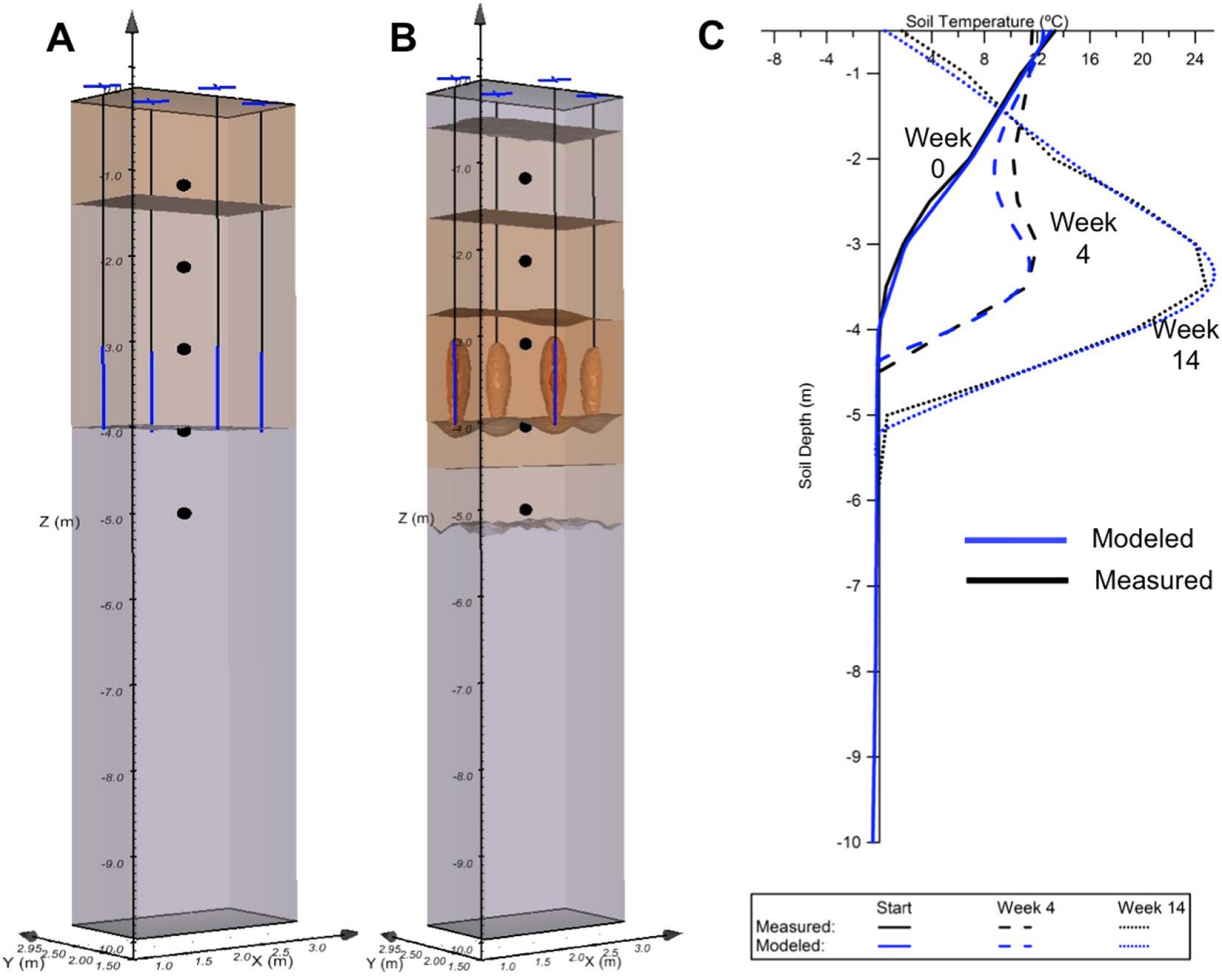
Thermal modeling results and comparison to borehole thermistor data. Panels A and B depict baseline (**A**) and final 3D thermal states (**B**) as predicted by a finite-element thermal transfer simulator for a small sub-section of the heater plot surrounding monitoring well BH14. Panel C shows a comparison between the modeled (blue lines) and measured (black lines) thermal profiles at BH14 for weeks 0, 4 and 14.

To further evaluate the likely 3D temperature distribution of the site, a commercial finite-element thermal transport code (SVOffice5, SoilVision Systems Ltd.) was used to model a small portion of the heater array. We assumed a homogeneous soil column with a dry soil density of 800 kg/m^3^, a thermal conductivity of 0.55 W/mK, and a 40% volumetric water content, broadly consistent with soil texture analyses conducted on archival core. Four heaters surrounding BH14 were modeled using known temperatures at the heater elements as Dirichlet boundary conditions. Surface temperature boundary conditions were also imposed to capture seasonal cooling effects. Figure 6 depicts the modeling results for both the baseline state (panel A) and the thermal state at 14 weeks (panel B). Panel C shows the predicted and measured temperature profile at baseline, 4, and 14 weeks. As can be seen, the measured data were closely matched given these relatively simplistic assumptions, which did not include advective heat transport, nor geomechanical processes (e.g. subsidence). Most importantly, the predicted permafrost table ablation is reasonably uniform between heaters despite thermal gradients in the unfrozen soil column.

Several advantages of the deep warming approach should be mentioned. While the areal energy flux (~55 W/m^2^) used in this study is substantial, this relatively large input allows completion of subsidence studies in short periods, compatible with summer arctic field deployments. The use of borehole heaters installed at the permafrost table also enables energy to be directly delivered to the region of interest rather than heating a much larger volume of soil from the surface; a similar heating experiment using IR lamp arrays would have required a dramatically higher power consumption.

A final advantage to the instrumented deep thawing approach is the combination of control of input energy, measurement of internal/surface temperature boundary conditions, and spatial quantification of deformation, parameters required for validating coupled THM models currently under development. A variety of approaches have been considered^22–24,42–44^ but no field validation datasets at the decameter scale are available. As a result, validation often relies on a combination of analytical solutions, laboratory studies, and field measurement which often lack constraints on energy fluxes. We view this intermediate scale of thaw deformation test as a transformational step in confirming the validity of new solution schemes and permafrost constitutive models. We should note that the higher subsurface temperatures generated during rapid thaw experiments would complicate biogeochemical studies due to both the thermal scaling of chemical kinetics and rapid shifts in microbial population to a mesophilic domain; neither of these problems are present for THM model calibration and monitoring validation.

In conclusion, the approach demonstrated in this study provides a path forward for testing the impact of a wide range of future climate scenarios on permafrost behavior. In particular, similar systems could be utilized to test novel monitoring approaches as well as the performance of resilient or adaptive civil infrastructure in a warming Arctic.

## Electronic supplementary material

Supplemental Information

## References

[1] Jorgenson, M. T., Shur, Y. L. & Pullman, E. R. Abrupt increase in permafrost degradation in Arctic Alaska. *Geophysical Research Letters* **33**, 10.1029/2005GL024960 (2006).

[2] Romanovsky VE, Smith SL, Christiansen HH (2010). Permafrost thermal state in the polar Northern Hemisphere during the international polar year 2007–2009: a synthesis. Permafrost and Periglacial processes.

[3] Schuur E (2015). Climate change and the permafrost carbon feedback. Nature.

[4] Nelson FE, Anisimov OA, Shiklomanov NI (2001). Subsidence risk from thawing permafrost. Nature.

[5] Kokelj S, Jorgenson M (2013). Advances in thermokarst research. Permafrost and Periglacial Processes.

[6] Schaefer K, Lantuit H, Romanovsky VE, Schuur EA, Witt R (2014). The impact of the permafrost carbon feedback on global climate. Environmental Research Letters.

[7] Kokelj SV, Lantz TC, Tunnicliffe J, Segal R, Lacelle D (2017). Climate-driven thaw of permafrost preserved glacial landscapes, northwestern Canada. Geology.

[8] Vincent WF, Lemay M, Allard M (2017). Arctic permafrost landscapes in transition: towards an integrated Earth system approach. Arctic Science.

[9] Chadburn S (2017). An observation-based constraint on permafrost loss as a function of global warming. Nature Climate Change.

[10] Ge, S., McKenzie, J., Voss, C. & Wu, Q. Exchange of groundwater and surface‐water mediated by permafrost response to seasonal and long term air temperature variation. *Geophysical Research Letters* **38**, 10.1029/2011GL047911 (2011).

[11] Frohn RC, Hinkel KM, Eisner WR (2005). Satellite remote sensing classification of thaw lakes and drained thaw lake basins on the North Slope of Alaska. Remote sensing of environment.

[12] Harris, S. A. *The permafrost environment* (Barnes & Noble, 1986).

[13] Andersland, O. B. & Ladanyi, B. *Frozen ground engineering* (John Wiley & Sons, 2004).

[14] Fortier R, LeBlanc A-M, Yu W (2011). Impacts of permafrost degradation on a road embankment at Umiujaq in Nunavik (Quebec), Canada. Canadian Geotechnical Journal.

[15] Hayashi, M. The cold vadose zone: Hydrological and ecological significance of frozen-soil processes. *Vadose Zone Journal* **12**, 10.2136/vzj2013.03.0064 (2013).

[16] Melvin AM (2017). Climate change damages to Alaska public infrastructure and the economics of proactive adaptation. Proceedings of the National Academy of Sciences.

[17] Rowland, J., Travis, B. & Wilson, C. The role of advective heat transport in talik development beneath lakes and ponds in discontinuous permafrost. *Geophysical Research Letters* **38**, 10.1029/2011GL048497 (2011).

[18] Atchley AL (2015). Using field observations to inform thermal hydrology models of permafrost dynamics with ATS (v0. 83). Geoscientific Model Development.

[19] Grosse G, Goetz S, McGuire AD, Romanovsky VE, Schuur EA (2016). Changing permafrost in a warming world and feedbacks to the Earth system. Environmental Research Letters.

[20] Wainwright HM (2015). Identifying multiscale zonation and assessing the relative importance of polygon geomorphology on carbon fluxes in an Arctic tundra ecosystem. Journal of Geophysical Research: Biogeosciences.

[21] McKenzie JM, Voss CI, Siegel DI (2007). Groundwater flow with energy transport and water–ice phase change: numerical simulations, benchmarks, and application to freezing in peat bogs. Adv Water Resour.

[22] Rempel, A. W. Hydromechanical processes in freezing soils. *Vadose Zone Journal* **11**, 10.2136/vzj2012.0045 (2012).

[23] Uchida, S., Soga, K. & Yamamoto, K. Critical state soil constitutive model for methane hydrate soil. *Journal of Geophysical Research: Solid Earth* **117**, 10.1029/2011JB008661 (2012).

[24] Bense, V., Kooi, H., Ferguson, G. & Read, T. Permafrost degradation as a control on hydrogeological regime shifts in a warming climate. *Journal of Geophysical Research: Earth Surface* **117**, 10.1029/2011JF002143 (2012).

[25] Hubbard SS (2013). Quantifying and relating land-surface and subsurface variability in permafrost environments using LiDAR and surface geophysical datasets. Hydrogeology Journal.

[26] Walvoord, M. A. *et al*. Improved Understanding of Permafrost Controls on Hydrology in Interior Alaska by Integration of Ground-Based Geophysical Permafrost Characterization and Numerical Modeling. (U.S. Geological Survey, Final Report for Department of Defense Strategic Environmental Research and Development Program (SERDP), 2015).

[27] Walvoord, M. A. & Kurylyk, B. L. Hydrologic impacts of thawing permafrost—A review. *Vadose Zone Journal* **15**, 10.2136/vzj2016.01.0010 (2016).

[28] Liu L (2015). Remote sensing measurements of thermokarst subsidence using InSAR. Journal of Geophysical Research: Earth Surface.

[29] Briggs MA (2017). Surface geophysical methods for characterising frozen ground in transitional permafrost landscapes. Permafrost and Periglacial Processes.

[30] Dafflon B (2016). Geophysical estimation of shallow permafrost distribution and properties in an ice-wedge polygon-dominated Arctic tundra region. Geophysics.

[31] Beyens L, Ledeganck P, Graae B, Nijs I (2009). Are soil biota buffered against climatic extremes? An experimental test on testate amoebae in arctic tundra (Qeqertarsuaq, West Greenland). Polar biology.

[32] Hinkel KM, Hurd JK (2006). Permafrost destabilization and thermokarst following snow fence installation, Barrow, Alaska, USA. Arctic, Antarctic, and Alpine Research.

[33] Natali SM (2011). Effects of experimental warming of air, soil and permafrost on carbon balance in Alaskan tundra. Global Change Biology.

[34] Natali SM, Schuur EA, Webb EE, Pries CEH, Crummer KG (2014). Permafrost degradation stimulates carbon loss from experimentally warmed tundra. Ecology.

[35] Natali SM, Schuur EA, Rubin RL (2012). Increased plant productivity in Alaskan tundra as a result of experimental warming of soil and permafrost. Journal of Ecology.

[36] Hanson PJ (2011). A method for experimental heating of intact soil profiles for application to climate change experiments. Global Change Biology.

[37] Hanson PJ (2017). Attaining whole-ecosystem warming using air and deep-soil heating methods with an elevated CO_2_ atmosphere. Biogeosciences.

[38] Krassovski M (2015). A comprehensive data acquisition and management system for an ecosystem-scale peatland warming and elevated CO_2_ experiment. Geoscientific Instrumentation, Methods and Data Systems.

[39] Wagner, A. M., Beede, M. C. & Zufelt, J. E. Permafrost Ecosystem Warming Prototype: Installation, Operation, and Initial Site Characterization. (ERDC/CRREL TR-13-13, Engineer Research and Development Center, Cold Regions Research and EngineeringLaboratory, NH, 2013).

[40] Shulski, M. & Wendler, G. *The climate of Alaska* (University of Alaska Press, 2007).

[41] Linell, K. A. Long-term effects of vegetative cover on permafrost stability in an area of discontinuous permafrost. In *2nd International Conference on Permafrost*, 688-693 (National Academy of Sciences).

[42] Sjöberg Y (2016). Thermal effects of groundwater flow through subarctic fens: A case study based on field observations and numerical modeling. Water Resources Research.

[43] Wettlaufer J, Worster MG (2006). Premelting dynamics. Annu. Rev. Fluid Mech..

[44] McKenzie JM, Voss CI (2013). Permafrost thaw in a nested groundwater-flow system. Hydrogeology Journal.

