# Supplementary material for: Permafrost Degradation and Subsidence Observations during a Controlled Warming Experiment
Source: Sci Rep. 2018 Jul 19;8:10908. doi: 10.1038/s41598-018-29292-y (PMC6053422; doi:10.1038/s41598-018-29292-y)
Supplement: Supplementary file 1 — Supplemental Information [file 41598_2018_29292_MOESM1_ESM.docx]

**Supplementary Information for:**

**Permafrost Degradation and Subsidence Observations during a Controlled Warming Experiment**

Anna M. Wagner^1^, Nathaniel J. Lindsey^2,3^, Shan Dou^3^, Arthur Gelvin^1^, Stephanie Saari^1^, Christopher Williams^4^, Ian Ekblaw^3^, Craig Ulrich^3^, Sharon Borglin^3^, Alejandro Morales^3^, Jonathan Ajo-Franklin^3*^

1. U.S. Army Cold Regions Research & Engineering Laboratory (CRREL), Fairbanks, AK

2. Earth and Planetary Sciences Department, University of California, Berkeley, CA

3. Lawrence Berkeley National Laboratory, Berkeley, CA

4. U.S. Army Cold Regions Research & Engineering Laboratory (CRREL), Hanover, NH

* Correspondence to JBAjo-Franklin@lbl.gov

Soil Texture Analysis

To evaluate soil texture characteristics at the site, small samples were obtained from archival cores P2 (center of heated zone) and P6 (northwest of heated zone). Sub-samples were weighed, dispersed in Sodium Hexametaphosphate, and analyzed using a laser particle size analyzer (Malvern Instruments Inc, Mastersizer 3000). Depths between 4.04 m and 8.18 m were sampled, corresponding to the primary thaw zone. As can be seen in Table S1, all samples are classified as Silt loams under USDA soil classification guidelines with the majority of each sample by volume consisting of particles between 2 and 50 microns (silt). Clay fractions were surprisingly small (< 1% by volume) and the sand fractions were dominated by very fine material (50-100 micron). Figure S1 shows the results on the US Department of Agriculture: Natural Resources Conservation Services soil triangle. In summary, the analyzed samples are consistent with the relatively homogeneous wind-blown Fairbanks silt deposits common in the region.

Table S1 : Grain size and soil texture results from P2 and P6 cores as obtained by laser particle size analysis.


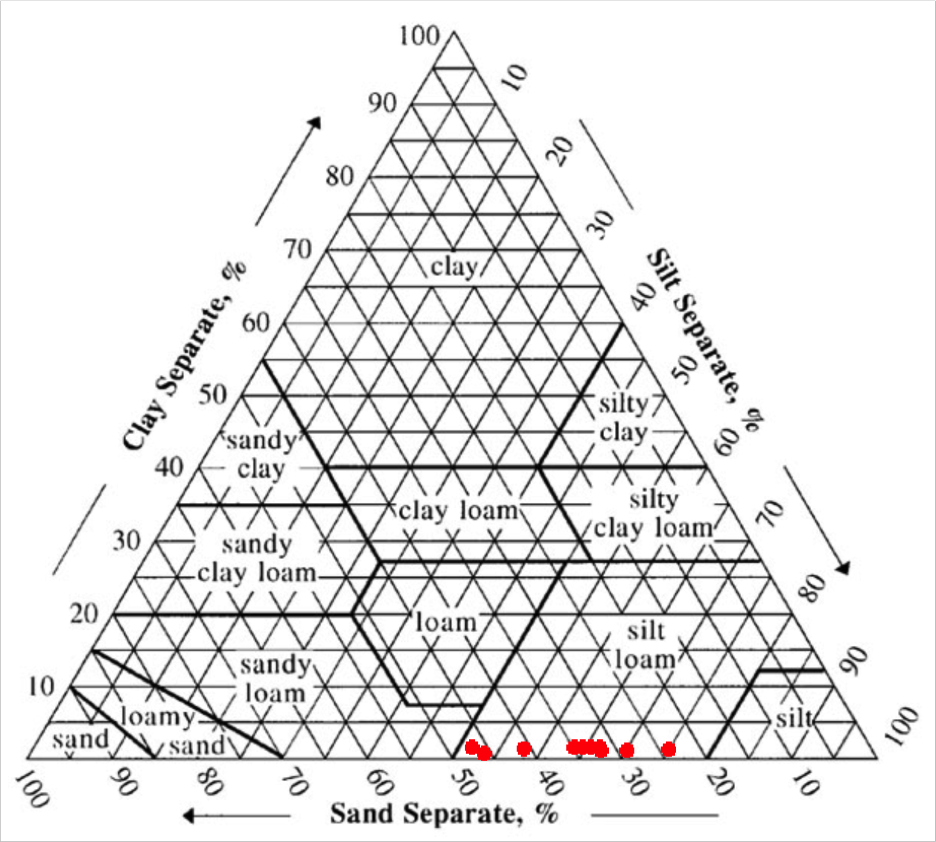


Figure S1 : Grain size analysis for samples obtained from core locations P2 and P6. All soil analyzed were silt loams. Background soil triangle image courtesy of the US Department of Agriculture : Natural Resources Conservation Service.

**Cryotexture of permafrost as revealed by X-ray CT**

To better understand dominant cryotexture and ice distribution, core was obtained for location OP4, to the east of the thaw experiment plot. X-ray computed tomography (CT) of extracted cores was conducted at Lawrence Berkeley National Laboratory. The scanning was performed using a modified medical GE Lightspeed scanner, with 16 slices using an energy of 120 kV and a current of 160 mA. Attenuation data from the CT scan is converted to Hounsfield Units which are correlated to density through calibration obtained by scanning known density objects. Each voxel from the scan was 625 micron dimension oriented in the axial direction, and depending on the sample the voxel in the radial dimensions ranged from 193 to 250 microns, depending on sample diameter. To prevent thawing, samples were kept cold until placed in the CT scanner. As can be seen in Fig. S2, ice distribution was laminar with several zones of higher ice fraction. Figure S2 panels A and B show central slices of sequential cores acquired through the permafrost table and below with blue colors corresponding to lower density zones of ice rich material. Panel C shows depth slices of the core shown in panel B.

**
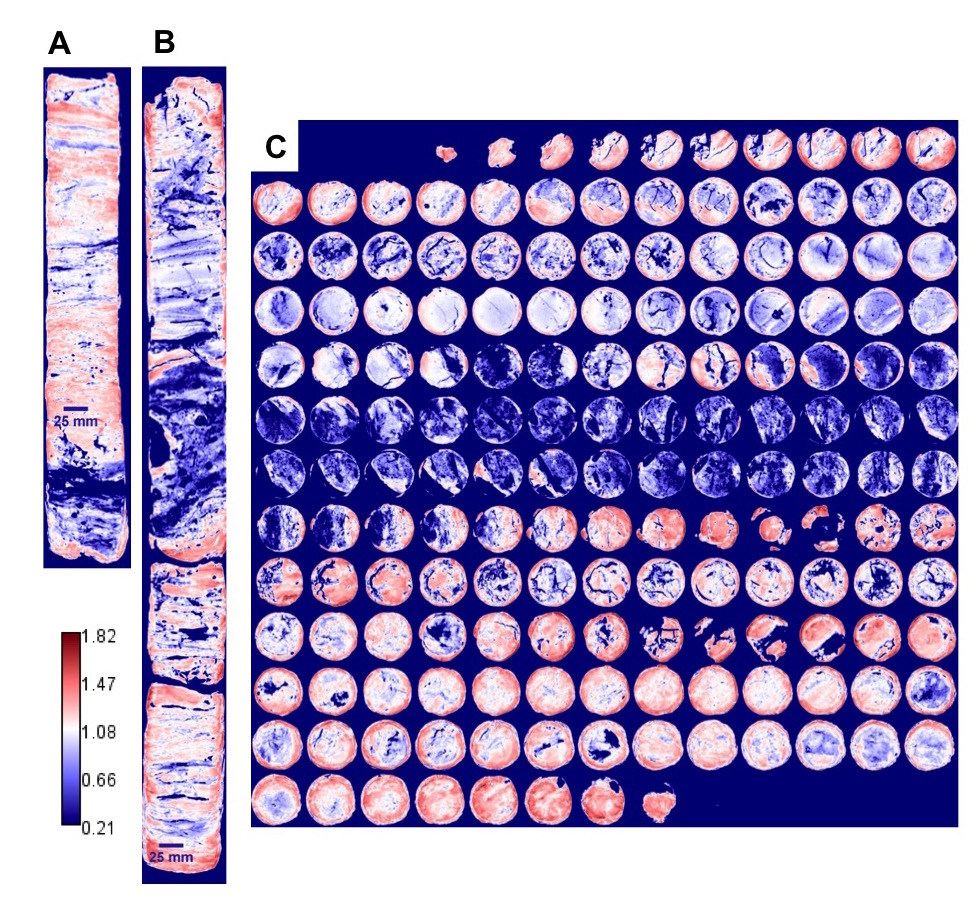
**

Figure S2 : CT scans of permafrost cores from location OP4. Panels A and B are length-oriented slices of depth sequential cores. Panel C shows depth slices of the core section in panel B.

**Heater stability and energy usage**

As mentioned in the primary manuscript, the heater array proved reliable over the 14 week duration of the experiment, depicted below in Fig. S3. While the total measured draw of the heating system ranged between 6.6 and 6.8 kW, diurnal fluctuations were observed, likely tied to fluctuations in the power grid. The heating system was operated in close to constant energy flux mode rather than at a constant borehole temperature; as a result, heater casing temperature increased over the duration of the experiment, peaking near 45 ^o^C before termination of the experiment. Diurnal fluctuations in energy input yielded small variations in heater casing temperatures (< 0.2 ^o^C) as well which were not observed in any interwell measurements.


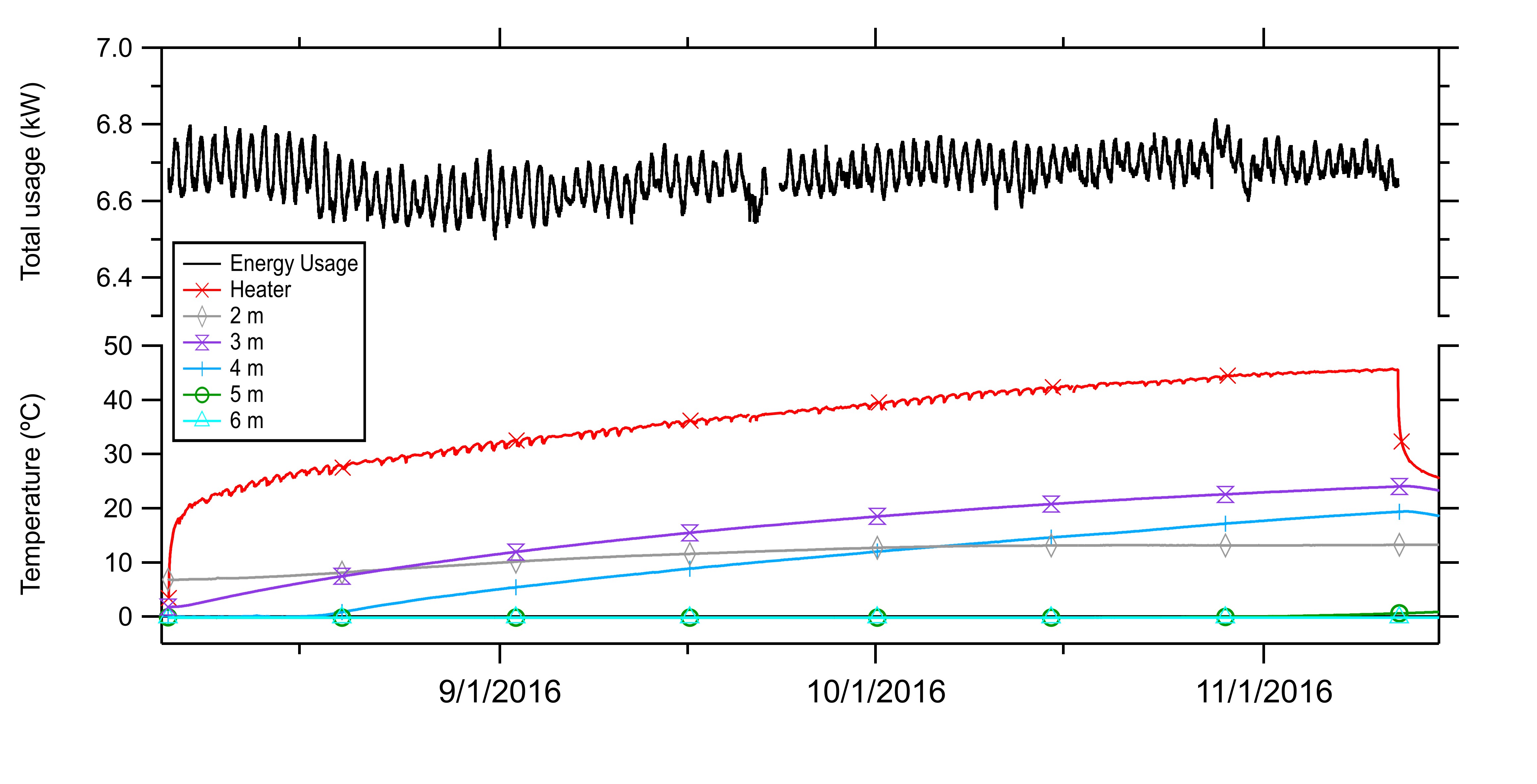


Figure S3 : Heater stability and casing temperatures : The top panel depicts total power usage of the heater array over the duration of the experiment. The bottom panel shows thermocouple measured temperature at a single heater as well as temperature in the interwell region (thermistor well BH14) over the same time duration.

**Off-plot Heating Signatures**

During the heating experiment, permafrost thaw was not observed in upslope thermal measurements. Figure S4 shows long-term borehole thermistor datasets from one on-plot measurement borehole (BH14), a second located to the east off of the heater array (BH16) in a zone of shallower permafrost, and a third downslope to the west (BH13) in a zone of deeper permafrost. At 2 m depth, seasonal variations are visible for both BH14 and BH13 while BH16 remains slightly below 0 ^o^C. BH14 also measures the heater pulse at 2 m, superimposed on the seasonal cycle. At 4 m, the thermal pulse from the heating is quite strong on the plot (BH14) and is also weakly observed via advective transport downslope off of the plot at BH13 (center panel). BH16 (upslope, off plot) remained below 0 ^o^C during the experiment. At 6 m depth, all three locations remain below 0 ^o^C.


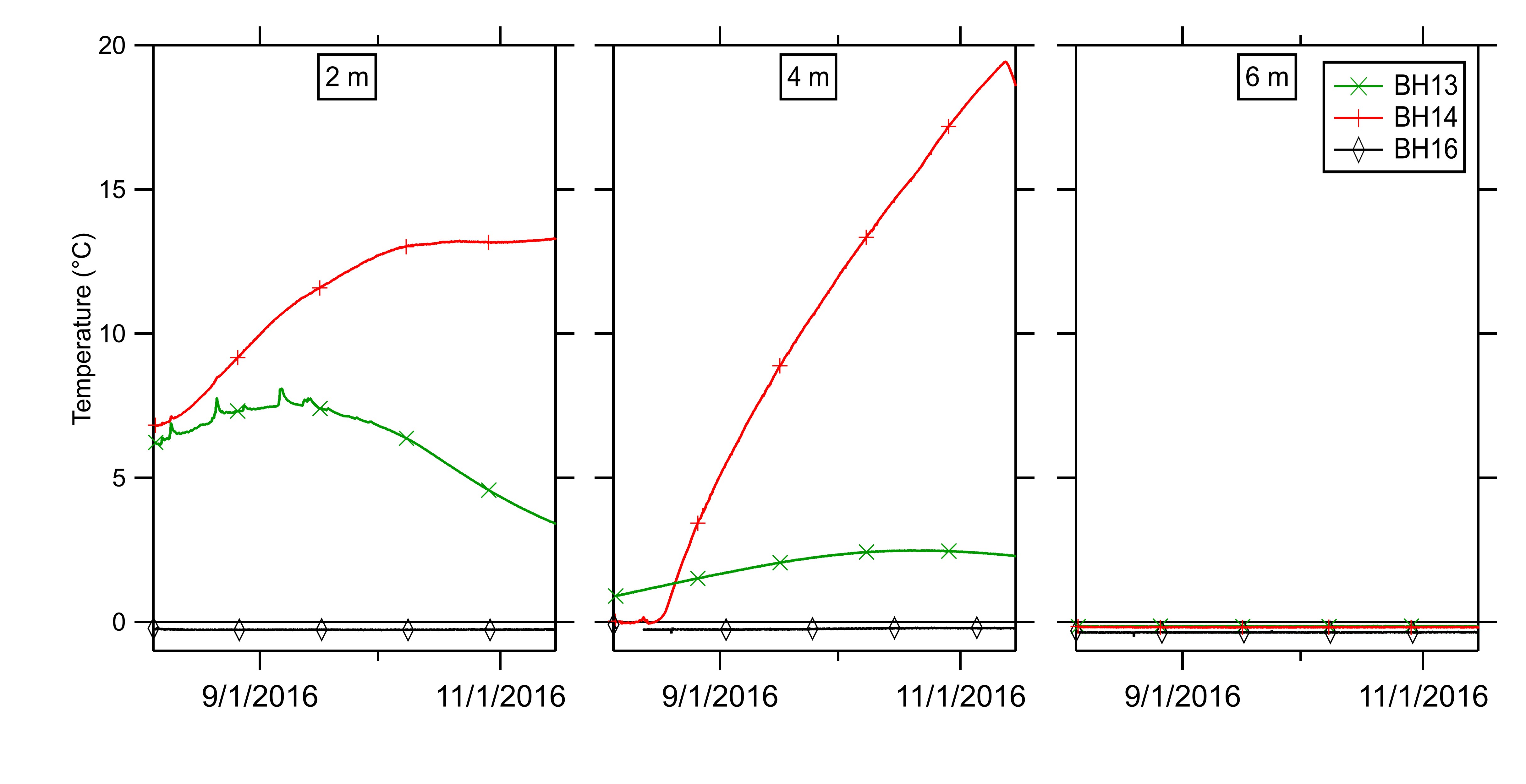


Figure S4 : Temperature histories for on and off-plot thermistor monitoring locations at 2 (left), 4 (center), and 6 (right) meter depths.
